# Cell Behavior of Non-Small Cell Lung Cancer Is at EGFR and MicroRNAs Hands

**DOI:** 10.3390/ijms222212496

**Published:** 2021-11-19

**Authors:** Sarah Sayed Hassanein, Sherif Abdelaziz Ibrahim, Ahmed Lotfy Abdel-Mawgood

**Affiliations:** 1 Biotechnology Program, Basic and Applied Sciences (BAS) Institute, Egypt-Japan University of Science and Technology (E-JUST), Alexandria 21934, Egypt; ahmed.mawgood@ejust.edu.eg; 2 Department of Zoology, Faculty of Science, Cairo University, Giza 12613, Egypt; isherif@sci.cu.edu.eg

**Keywords:** epidermal growth factor receptor (EGFR), microRNA (miRNA), therapeutic targets, diagnostic markers, signaling pathways, oncogenes, oncosuppressors, chemoresistance, tyrosine kinase inhibitors (TKIs), non-small cell lung cancer (NSCLC)

## Abstract

Lung cancer is a complex disease associated with gene mutations, particularly mutations of Kirsten Rat Sarcoma Viral Oncogene Homolog (*KRAS*) and epidermal growth factor receptor (*EGFR*). Non-small cell lung cancer (NSCLC) and small cell lung cancer (SCLC) are the two major types of lung cancer. The former includes most lung cancers (85%) and are commonly associated with EGFR mutations. Several EGFR-tyrosine kinase inhibitors (EGFR-TKIs), including erlotinib, gefitinib, and osimertinib, are effective therapeutic agents in *EGFR*-mutated NSCLC. However, their effectiveness is limited by the development (acquired) or presence of intrinsic drug resistance. MicroRNAs (miRNAs) are key gene regulators that play a profound role in the development and outcomes for NSCLC via their role as oncogenes or oncosuppressors. The regulatory role of miRNA-dependent *EGFR* crosstalk depends on *EGFR* signaling pathway, including Rat Sarcoma/Rapidly Accelerated Fibrosarcoma/Mitogen-Activated Protein Kinase/Extracellular Signal-Regulated Kinase 1/2 (Ras/Raf/MEK/ERK1/2), Signal Transducer and Activator of Transcription (STAT), Nuclear Factor Kappa-Light-Chain-Enhancer of Activated B Cells (NF-kB), phosphoinositide 3-kinase/protein kinase B (PI3K/AKT), Janus kinase 1 (JAK1), and growth factor receptor-bound protein 2 (GRB2). Dysregulated expression of miRNAs affects sensitivity to treatment with EGFR-TKIs. Thus, abnormalities in miRNA-dependent EGFR crosstalk can be used as diagnostic and prognostic markers, as well as therapeutic targets in NSCLC. In this review, we present an overview of miRNA-dependent EGFR expression regulation, which modulates the behavior and progression of NSCLC.

## 1. Introduction

**Figure 1:**
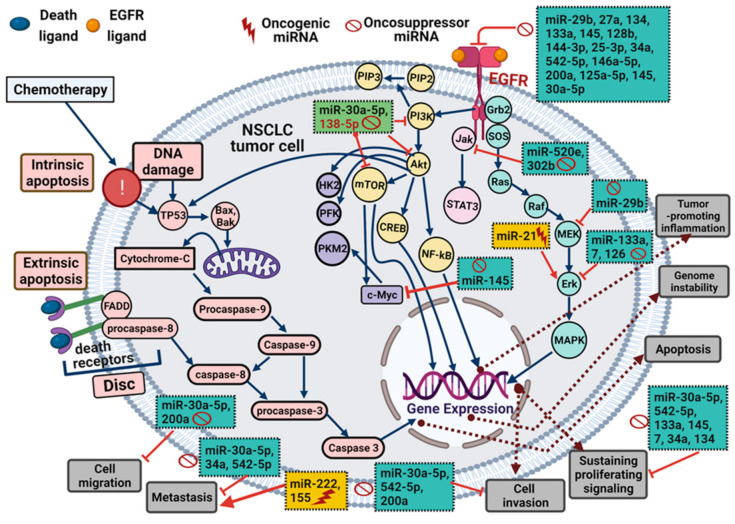
EGFR signaling components are affected by various miRNAs (oncogenic or tumor suppressors) in non-small cell lung cancer. Activation of the EGFR signaling pathway, including PI3K/Akt, Ras/Raf/MAPK, and Jak/STAT, stimulates inflammation, proliferative signaling, migration, angiogenesis, and invasion. These signaling pathways are controlled by different miRNAs. Binding of death ligand (e.g., TRAIL) to death receptor leads to FADD (adaptor molecule). Pro-caspase-8 activation takes place upon its binding to FADD and DISC formation (extrinsic apoptosis). Chemotherapeutic drugs, such as cisplatin, cause DNA damage and results in p53 activation (intrinsic apoptosis). Activated caspase-8 directly activates other caspases that translocate to the mitochondria promoting the Bax-Bak assembly, thus changing mitochondrial membrane permeability. Cytochrome c is then released into cytosol resulting in caspases activation leading to apoptosis. Several oncogenic and tumor suppressor miRNAs control EGFR signaling components and subsequently affect tumor growth and progression. Blue and red arrows for stimulation, dashed brown arrows for cellular effect, and red “T” for inhibition.

Globally, lung cancer is the foremost cancer type in terms of incidence and mortality rates in men and women [1,2,3]. It is classified into small cell lung cancer (SCLC) and non-small cell lung cancer (NSCLC), with approximately 15% and 85% of lung cancer cases, respectively [4,5,6]. Despite progress in treatment strategies of NSCLC, the overall five-year survival rate is still poor due to late diagnosis and drug resistance development, particularly to tyrosine kinase inhibitors (TKIs) [2,7,8,9]. Receptor tyrosine kinases (RTKs), namely epidermal growth factor receptor (*EGFR*), are commonly overexpressed in NSCLC and associated with poor prognosis [10,11]. The signaling pathway of *EGFR* activates intracellular signaling cascades, including Signal Transducer and Activator of Transcription (STAT), Rat Sarcoma/Rapidly Accelerated Fibrosarcoma/Mitogen-Activated Protein Kinase/Extracellular Signal-Regulated Kinase 1/2 (Ras/Raf/MEK/ERK1/2), phospholipase C (PLC)/protein kinase C (PKC), and phosphoinositide 3-kinase/phosphoinositide 3-kinase/Protein kinase B (PI3K/Akt) pathways that boost angiogenesis, tumor cell proliferation, metastasis, invasion, and apoptosis evasion [10,12,13,14,15,16] (Figure 1). In lung cancer cells, the expression of EGFR and their ligands, especially transforming growth factor-alpha (TGFα), signifies the presence of a self-stimulatory (autocrine) growth factor loop [17]. For in-depth molecular signaling pathways of EGFR, we refer the readers to recent reviews [11,18].

MicroRNAs (miRNAs) are known as small non-coding RNAs consisting of 18–25 nucleotides and are nodal post-transcriptional gene regulators [19,20,21]. Individual miRNA plays a key role in targeting and regulating many mRNAs expression either by promoting mRNAs degradation or inhibiting their translation. Thus, individual mRNA can be regulated by several miRNAs [15,22,23,24]. Aberrant miRNAs expression patterns can be used as diagnostic and prognostic markers in NSCLC [4,8,25,26,27,28]. MiRNA-mRNA interactions are associated with many biological hallmarks of lung cancer [24,29,30]. Dysregulated expression of miRNAs represents a hallmark of several human cancers, including lung cancer, acting as oncogenes or tumor suppressors [5,20,21,31,32]. EGFR signaling pathways and miRNAs are key players in the development of NSCLC [31,33,34,35,36]. The dual role of miRNAs, either as oncogenes or oncosuppressors, has been revealed in different studies, and that basically depends on their direct targets and the activated downstream signaling pathways [37,38,39,40]. Interestingly, the transduction of bronchial epithelial BEAS-2B cells with retroviral vectors expressing KRAS^G12V^ (Kirsten Rat Sarcoma Viral Oncogene Homolog) and monitoring miRNA expression patterns by microarray analysis was performed to identify miRNAs implicated in EGFR signaling in NSCLC patients. This approach could define miR-29b as an important target for upregulation by mutant *KRAS*, wherein the pharmacologic inhibition of EGFR or MEK was sufficient to reduce miR-29b expression levels. Anti-miR-29b constructs increased apoptosis sensitivity, implying that mutant *KRAS* conferred apoptotic resistance by miR-29b [41,42,43]. The ability of miR-29b to mediate this effect was ascribed to targeting TNFAIP3/A20, a negative regulator of NF-κB signaling. Overexpression of a miR-29b–refractory isoform of TNFAIP3 restored NF-κB and extrinsic apoptosis, proving that TNFAIP3 is a functionally important target for miR-29b. Further, miR-29b confers sensitivity to intrinsic apoptosis induced by cisplatin exposure. Thus, miR-29b expression can cause cells to shift from extrinsic to intrinsic apoptosis mechanisms. From these data, miR-29b can act as an oncogene or a tumor suppressor gene, depending on the signaling context [41,43,44,45,46]. In the next sections, we comprehensively discuss the reciprocal regulation of miRNAs and EGFR expression and activation and how this regulation affects NSCLC behavior.

## 2. EGFR Overexpression/Hyperactivation and miRNA Expression Pattern: Effects on NSCLC Cell Behavior

### 2.1. Effect of miRNAs on EGFR Expression

Emerging data suggest that *EGFR* mRNA is overexpressed in a variety of human tumors, including NSCLC, and is involved in tumor growth [47,48,49,50,51]. The behavior of lung cancer cells is associated with miRNA-dependent EGFR expression (Table 1). For instance, EGFR and c-MET (receptor for hepatocyte growth factor) are implicated in various cellular processes and regulated by many miRNAs, leading to tumor progression [52,53,54]. A biological relationship between *EGFR, MET*, and the miRNA cluster 23a∼27a∼24–2 was unveiled, where miR-27a regulated *MET, EGFR*, and Sprouty2 in a panel of NSCLC cell lines (293, 293TN, H460, A549, H1299). According to these findings, miR-27a could down-regulate *MET* and *EGFR* by targeting their 3′ UTRs directly or indirectly through Sprouty2; consequently, the underlying mechanism of the *MET* and *EGFR* axis regulation may emerge as new strategies in lung cancer treatment [52,53]. *EGFR* was also a direct target of miR-134, evidenced by luciferase assays, and the overexpression of miR-134 suppressed *EGFR* expression in NSCLC cells [36,55,56]. Likewise, *EGFR* was a direct target of miR-34a, and the siRNA knockdown of EGFR inhibited cell proliferation, promoted apoptosis, and suppressed cell cycle progression. Using quantitative real-time PCR (qRT-PCR) analysis, miR-34a expression was significantly reduced in carcinoma tissues and cell lines of NSCLC, implying that miR-34a can be a tumor suppressor in lung cancer. Furthermore, in both the A549 xenograft model and metastatic tumors in nude mice, miR-34a inhibits tumor development [57,58,59,60,61,62,63].

A mRNA-miRNA stepwise regression model and database-dependent miRNA target prediction were performed to identify the miRNA-dependent EGFR signaling cascade. This model revealed that the EGFR ligand EGF is positively correlated with circulating miR-145 and miR-199a-5p in the pre-surgery and post-surgery NSCLC patients, respectively. Unexpectedly, miR-495 was positively related to PTK2 in both groups [4]. Thus, it can be inferred that miRNAs can regulate different components of the EGFR signaling pathway, namely ligand, receptor, or the downstream signaling molecules [10,12,13,14,15,16,22,23,24]. In this regard, the expression of miR-133a in NSCLC tissue was found to be lower than in adjacent mucosae. Furthermore, as compared to the adjacent mucosae, NSCLC tissue had higher *EGFR* expression. Besides, the upregulation of miR-133a inhibited cell growth and induced apoptosis in NSCLC cells, which in turn increased caspase-3 protein expression, while suppressing EGFR, phosphorylated (p)-AKT, and phosphorylated (p)-ERK (Figure 1). These findings shed light on the function of miR-133a and the molecular mechanisms underlying miR-133a-mediated EGFR/AKT/ERK signaling pathway downregulation in NSCLC [64,65,66]. Further, miR-145 significantly suppressed *EGFR* expression and inhibited cancer cell growth compared with negative control miRNA in A549 NSCLC cells [7,83,84]. Transfection with miR-146a mimic in NSCLC cells resulted in the downregulation of *EGFR* mRNA. Meanwhile, inverse results were observed when transfected with miR-146a inhibitor. Furthermore, high miR-146a expression showed longer progression-free survival in tissues of NSCLC patients. As a result, miR-146a is a potent prognostic biomarker in NSCLC [85,86,87]. Moreover, the expression levels of miR-128b were lower in NSCLC carcinoma tissues than in adjacent non-neoplastic tissues, whereas the expression of *EGFR* mRNA was the opposite. According to immunohistochemical staining analysis, normal tissues did not express EGFR protein, and cancer tissues exhibited a 60% positive staining for EGFR. Furthermore, the relative expression of miR-128b levels was negatively correlated with *EGFR* mRNA and protein levels [49,88,89,90,91,92].

MiR-7 is an important modulator of EGFR-mediated oncogenesis [81,82,93,94]. Knockdown of *EGFR* mediated by short hairpin RNA (shRNA)–bearing lentiviruses induced enlarged cell size and growth arrest in CL1-5 lung cancer cells. MiRNA microarray analysis for these *EGFR*-silenced cells showed a significant downregulation of miR-7 confirmed by both qRT-PCR analysis and the RNase protection assay. In contrast, expression of the *EGFR* mutant (L858R), which enhanced phosphorylation of c-Myc and EGFR, promoted the expression of miR-7 in CL1-5 cells. These data indicated that miR-7 is induced by EGFR signaling via a Ras/ERK/Myc pathway [82,95,96] (Table 1). On the contrary, Zhang et al. reported the tumor suppressor effect of circular RNA ciRS-7 (CDR1as)/miR-7 signals. CDR1as levels significantly increased with the development of NSCLC cells and tissues, which was inversely correlated with miR-7 expression. The CDR1as overexpression resulted in increased cell vitality and development, which could be reversed by knockdown *CDR1as* or overexpressing miR-7, inducing apoptosis and G1/S arrest. CDR1as acted as miR-7 sponges, allowing up-regulation of direct miR-7 target genes, such as *EGFR, CCNE1,* and *PIK3CD*. The findings in vivo further indicated that CDR1as acted as an oncogene, up-regulating the proliferation index Ki-67, EGFR, CCNE1, and PIK3CD levels, thus inhibiting the anti-tumor effects of tumor suppressor miR-7 [94,95,96,97]. This is supported by the finding of decreased miR-7 expression quantified by qRT-PCR in murine Lewis lung cancer (3LL) cells [81]. Further, miR-7 expression restoration in vitro induced cell apoptosis, prevented 3LL cell proliferation, and decreased tumorigenesis in vivo. Using qRT-PCR and Western blot analysis, miR-7 has been shown to suppress the expression of both oncogenes, the murine leukemia viral oncogene homolog-1 (RAF-1), a downstream effecter of EGFR signaling, and EGFR [94,95,96,97]. Furthermore, in 3LL cells, EGFR inhibition had similar effects of transfection with miR-7 mimic. These data revealed that miR-7 functions as an anti-tumor miRNA in 3LL cells by targeting the expression of the oncogenes *EGFR* and *RAF-1* [81].

A study by Giordano et al. analyzed the expression of RNA in NSCLC tissues, including small RNAs using nCounter System^®^ (NanoString Technologies) to better understand the molecular features of young and old lung adenocarcinoma (LADC) patients. Seven miRNAs were found to be differentially expressed in the two groups; miR-25-3p, miR-33a-5p, miR-29c-3p, miR-153-3p, miR-144-3p, miR-342-5p, and miR-485-3p. The expression levels of these miRNAs were higher in NSCLC tissues of young patients than in older counterparts. Their predicted genes include *EGFR, MET, VEGF-A, TP53,* and *PDGFRa*. MiR-144-3p had the opposite effect on overall survival, where its upregulation was linked to a worse prognosis in young patients and a better prognosis in older patients [26,98,99,100]. Furthermore, an enrichment analysis explored 33 aberrantly expressed miRNAs in LADC and LSCC, of which miR-25-3p was a prospective prognostic biomarker in NSCLC by regulating TGFβ and EGFR signaling [8,101,102,103,104,105]. It has been recently reported that six differentially expressed miRNAs, including miR-31-5p, -5p, miR-708-5p, miR-451a, miR-30a-5p, and miR-126-3p, were notably associated with overall survival [9,70,106,107,108,109,110]. High expression levels of miR-31 and miR-21 were associated with poor prognosis, but better and prolonged survival was linked with elevated expression of miR-126, miR-708, miR-30a, and miR-451 in NSCLC patients [109,110]. The expression analysis and miRNA-hub gene network identified EGFR, phosphatase tensin homolog (PTEN), STAT3, vascular endothelial growth factor-A (VEGFA), transforming protein RhoA (RHOA), catenin beta 1 (CTNNB1), T53, and KRAS as possible target genes for these six miRNAs [9,70,106,107,108].

MiRNAs can modulate genes and pathways involved in lung tumorigenesis. It was reported that circulating a 3-miRNA signature, including miR-92a-3p, miR-16-5p, and miR-451a, had a high sensitivity (84%) and specificity (100%) to predict LADC and LSCC [27,106,111,112,113,114]. These miRNAs are expected to modulate EGFR, K-RAS, and PI3K/AKT signaling, implying that the 3-miRNA signature is biologically significant in LADC and LSCC [27]. Further, the correlation between a novel lncRNA (long non-coding RNA), *TRPM2-AS*, and the miR-138-5p/EGFR axis was investigated in NSCLC development. *TRPM2-AS* upregulation in NSCLC tumors and cell lines was validated and associated with induced cellular migration, proliferation, and invasion, as well as inhibiting cell apoptosis; TRPM2-AS was positively associated with EGFR but inversely correlated with miR-138-5p. PI3K/AKT/mTOR axis was stimulated by plasmid cloning DNA (pcDNA)-*EGFR* but deactivated by miR-138-5p mimics [115,116,117,118]. The downregulation of miR-542-5p was inversely correlated with EGFR protein expression, increased vascular invasion, advanced TNM stage, lymphatic metastasis, and poor prognosis in NSCLC (LADC and LSCC). In a CAM (chick chorioallantoic membrane) model, miR-542-5p mimic significantly reduced tumor growth and angiogenesis. The same study, using bioinformatics methods, revealed 457 potential target genes of miR-542-5p as crucial players in cancer-related pathways, such as the cAMP signaling pathway and morphine addiction, of which six overexpressed genes, including *PDE4B, GABBR1, PDE4C, ADCY1, ADCY6,* and *GIPR* from the cAMP signaling pathway in NSCLCs tissues [67,119,120,121,122]. Overall, these data showed that miRNA-regulated *EGFR* could differentially affect tumor growth, proliferation, invasion, and metastasis.

### 2.2. Effect of EGFR Activation on miRNA Expression

EGFR hyperactivation, common in lung cancers with poor prognoses, governs miRNA expression, mediates distinct gene regulatory pathways (Table 1). The correlation between EGFR activation and miR-145 expression in normal human lung epithelia cell line (BEAS-2B), LADC cell lines with wild-type *EGFR* (A549 and H292), and with mutant EGFR (H1975 and H1650) was investigated, where miR-145 levels were found to be strongly correlated with p-EGFR. EGF suppressed the expression of miR-145, particularly in BEAS-2B and A549 cells [7,83,84,123]. After inhibiting p-EGFR with AG1478, miR-145 was up-regulated, and treatment with AG1478 increased miR-145 by 67.5% in H1975 cells. Further, p-EGFR activated the ERK1/2 signaling pathway, and U0126 (ERK1/2 inhibitor) reversed the downregulation of miR-145 caused by EGFR activation [7,83,84]. Likewise, miR-134 suppressed EGFR-associated signaling by down-regulating p-EGFR in A549, H520 H1299, and H1975 cells. However, the down-regulation of p-Akt, p-STAT3, and p-ERK1/2 was not as concordant as predicted. H1299 cells showed decreased p-Akt and p-STAT3 but had no significant effect on p-ERK1/2; A549 cells showed decreased p-ERK1/2 and p-Akt but increased p-STAT3; H1975 cells exhibited increased p-STAT3 but decreased p-ERK1/2, whereas no significant changes in p-Akt; H520 cells showed decreased p-Akt, p-STAT3, and p-ERK1/2 [36,55,56]. Furthermore, overexpression of miR-134 inhibited EGFR-related signaling and reduced NSCLC cell proliferation by inducing apoptosis and/or cell cycle arrest, inferring that miR-134 acts as an oncosuppressor in NSCLC. The further mechanistic investigation, including rescue experiments and RNAi, indicated the downregulation of EGFR by miR-134 due to the anti-proliferative effect of miR-134. Moreover, in vivo experiments showed that miR-134 suppressed tumor growth of A549 xenograft in nude mice [36,55,56,124]. Immunoblotting showed that EGFR protein levels were also significantly downregulated with miR-7 downregulation [82,125,126,127,128].

**Figure 2:**
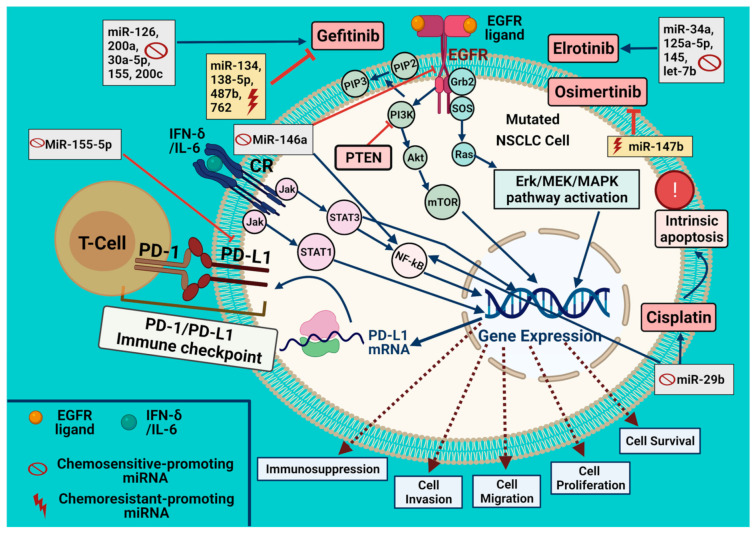
Role of miRNAs in modulating chemosensitivity to EGFR-TKIs and cisplatin in mutated NSCLC cells. In NSCLC tumor cells, EGFR activation results in activating Erk/MEK/MAPK, Akt/mTOR, and Jak/STAT signaling pathways. Cytokines (e.g., IFN-gamma and IL-6) bind to their respective cytokine receptors (CRs), leading to its activation. The NSCLC cell chemoresistance to gefitinib increases by miR-134, miR-138-5p, miR-487b, and miR-762 but decreases by miR-126, miR-200a, miR-30a-5p, miR-155, and miR-200c. The cell chemosensitivity to erlotinib was enhanced by miR-34a, miR-125a-5p, miR-145, and let-7b, yet it decreased to osimertinib by miR-147b. PD-L1 expression can be induced by aberrations in signal transduction components (constitutive expression) and/or many inflammatory cytokines (inducible expression); its expression results in its recruitment at the cell surface and binds to PD-1 on T-cell to avoid immune destruction. MiR-155-5p suppresses mRNA expression, membrane protein, and total protein levels of PD-L1. MiR-146a targets EGFR and NF-κB signaling and significantly suppresses cell proliferation via EGFR-TKI (erlotinib, gefitinib, and afatinib). MiR-29b restored NF-κB and extrinsic apoptosis. Cisplatin induces intrinsic apoptosis and can be repressed by miR-29b. Blue and red arrows denote for stimulation; dashed brown arrows for cellular effect and red “T” sign for inhibition.

Being expressed in T cells, B cells, macrophages, dendritic cells, and mesenchymal stem cells, programmed death ligand-1 (PD-L1 or B7-H1 or CD274) plays a key role in immune tolerance in tumors [129,130,131,132,133]. Previously, it was revealed that EGFR activation is due to PD-L1 overexpression in lung cancers, and PD-L1 expression level can be reduced by EGFR-TKIs [134,135,136,137,138]. Recently, the overexpression of miR-155-5p in LADC A549 cells was reported to suppress mRNA expression, membrane protein, and total protein levels of PD-L1 and significantly reduce IFN-c-induced PD-L1 expression [130,134,135,138,139]. These data indicate that miRNA can regulate EGFR indirectly to control inflammation and the immune response in lung cancer (Figure 2). Transfection of NSCLC cells with miR-146a mimic resulted in decreased EGFR protein levels and downstream signaling (ERK, AKT, and stat pathways) and these effects were reversed by miR-146a inhibitor treatment. Also, miR-146a induced cellular apoptosis, inhibited cell growth, and suppressed EGFR downstream signaling in NSCLC cell lines (H358, H1975, H1650, HCC827, and H292). MiR-146a also suppressed these NSCLC cells’ migration. These miR-146a-dependent effects are due to its targeting of NF-κB and EGFR signalings [85,86,87,140,141,142].

## 3. Specific miRNA Expression Patterns Affect the Behavior of *EGFR*-Mutated NSCLC

Emerging evidence indicated that miRNA–mRNA interactions regulate oncogenic processes involved in lung cancer in different ways depending on driver mutations in the tumor [31,33,35]. In NSCLC, somatic mutations in the TK domain of EGFR cause its constitutive activation along with its downstream signaling molecules through phosph-Akt even in the absence of ligand, leading to sustained proliferation, invasion and metastasis [143]. Upon tyrosine phosphorylation of the EGFR, recruitment of various signaling proteins, including the Nck (Nck Adaptor Protein), and Adaptor proteins GRB2 (Growth Factor Receptor-Bound Protein-2), SHC (Src Homology-2 Domain Containing Transforming Protein), PLC-γ (Phospholipase-C-γ), and STATs (Signal Transducer and Activator of Transcription) occurs [17,143,144,145,146,147,148,149,150]. *EGFR* mutation is a potent indicator of EGFR-TKIs efficiency in advanced NSCLC therapy, yet about 20–30% of *EGFR*-mutated cases exhibited no response to EGFR-TKIs, suggesting the presence of other factors independent of *EGFR* mutation [80,151,152,153]. The role of miRNAs (Table 1) in initial resistance to EGFR-TKIs in NSCLC with EGFR mutation is still mysterious. An integrative analysis showed the expression profile of miRNA–mRNA regulatory network in EGFR-mutated LADC using microarray for 155 LADC tissue specimens, where distinct 19 miRNA/431 mRNA signatures, with 5 miRNAs (miR-532-3p, miR-224-5p, miR-500a-3p, miR-532-5p, and miR-502-3p) were discovered as specific miRNAs for *EGFR*-mutated LADC [154]. The same study indicated 63 putative miRNA–mRNA interactions were potentially involved in *EGFR*-mutated tumor, including *DUSP4* and *MUC4* axes in *EGFR*-mutated LADC [154]. MiR-21 showed more dramatic expression changes in *EGFR*-mutated patient mutations as opposed to cases with *EGFR*-wild-type. In lung carcinoma cell lines, a strong association between p-EGFR and miR-21 levels and the suppression of miR-21 by the EGFR-TKI AG1478 indicates that EGFR signaling positively controls miR-21 expression. Antisense inhibition of miR-21 improved AG1478-induced apoptosis in the never-smoker-derived LADC cell line H3255, which had mutant EGFR and elevated levels of p-EGFR and miR-21. These findings indicate that abnormally elevated miR-21 expression, which is further enhanced by an active EGFR signaling system, plays a significant role in lung carcinogenesis and appears to be a possible therapeutic target in *EGFR*-mutant patients [86,155,156,157,158,159,160].

A large body of evidence has indicated the robust diagnostic accuracy of miRNAs in patients with mutated-*EGFR* versus wild-type *EGFR.* In accordance, the receiver operating curve unveiled the potential diagnostic value (AUC  =  0.81, *p*  <  0.0001) of circulating miR-504 in characterizing patients of *EGFR* exon 19 deletions from wild-type *EGFR* normalized to miR-191 [161]. Also, using a miRNA array profiling analysis, T790M/L858R-mutated lung cancer was found to have 20 up-regulated miRNAs, including miR-1 and miR-196a, compared to *EGFR* wild-type lung cancer [162]. Moreover, using Agilent microarrays, microRNA expression patterns were examined in 154 surgically resected LADC, and 20 matched normal lung tissue samples. Interestingly, 17 miRNAs were differentially expressed between *EGFR*-mutated and *EGFR* wild-type tumors, and 129 miRNAs were significantly differentially expressed in LADC compared with normal lung tissue [163]. In addition, miR-218 can negatively regulate *EGFR*, leading to decreased levels of p-STAT3 [164]. Besides, the downregulation of EGFR reduced the levels of p-STAT3 as well as cell viability more significantly in *EGFR*-mutated cells (H1975) than in *EGFR*-wild-type cells (A549) [165]. In addition, 12 miRNAs were substantially more or less abundant in *EGFR*-mutant patients, according to a class comparison study of miRNA expression between 6 *EGFR*-mutant cases and 22 *EGFR*-wild-type. MiRNAs (miR-21, miR-210, miR-486,miR-126, miR-126*, miR-138, miR-521, miR-451, miR-30d, and miR-30a) were altered in the same direction in cancer compared to non-neoplastic tissues, implying that *EGFR* mutations may reinforce the aberrant regulation of some miRNAs linked to lung carcinogenesis in never-smokers. MiR-21 (up-regulated) and miR-486 (down-regulated) were remarkable in malignant vs. non-cancerous tissues between *EGFR*-mutant and wild-type tumors [155]. Furthermore, a microarray study by Pak et al. investigated three alternately expressed miRNAs, including the tumor suppressor miR-34c, and oncogenes miR-183, and miR-210 in wild type and *EGFR*-mutated LADC (in exons 19 and 21), where miR-183 expression was notably higher in *EGFR*-mutated tissues than in wild-type tissues and positively correlated with lymphovascular invasion. Furthermore, the overexpression of miR-34c is associated with poor overall survival in *EGFR*-mutated (exon 19) group [25]. A 5-miRNA signature has been identified as predictive markers for *EGFR* mutation in NSCLC, namely miR-25, miR-122, miR-195, miR-21, and miR-125b in tumor tissues and plasma, where plasma levels of has-miR-125b expression in the patients were linked to disease-free survival, and overall survival, thereby acting as a prognostic marker [69]. Further, hsa-miR-126-3p, hsa-miR-21, hsa-miR-145, hsa-miR-183, hsa-miR-182, and hsa-miR-210 were found to be the most differentially expressed miRNAs in lung carcinoma tissues as compared to the adjacent normal lung parenchyma. Most intriguingly, after transfection of hsa-pre-miR-145, an apparent inhibition of cell growth was observed in the *EGFR-* mutant LADC [7,123].

In LADC tissues, a unique expression pattern of miRNAs was profiled for diverse oncogenic mutations such as KRAS-positive, *EGFR*-positive, and *EGFR*/*KRAS*-negative, in which miR-328, miR-20a, miR-18b, and miR-34c expression levels were highly up-regulated, while miR-32, miR-342, and miR-137 were downregulated [166]. miR-25, miR-155, and miR-495 were overexpressed in *EGFR*-positive, *EGFR*/*KRAS*-negative, and *KRAS*-positive LADCs groups, respectively. However, let-7g was down-expressed in all three groups, especially in *EGFR/KRAS*-negative LADC [166]. Kim et al. explored that miR-124, miR-1, and miR-196a were overexpressed in patients with *EGFR* T790M mutations and resistant to EGFR-TKI in NSCLC [162]. Diagnostic and prognostic classifiers were developed based on miRNAs expression levels as well as mutational gene status [155,162,163]. They showed great potential as potent and economic next-generation tools to enhance and complement current genetic tests. A diagnostic classifier was generated to show how miR-26a-5p, miR-504, and miR-1253 expression levels can classify NSCLCs such as mutant *EGFR*, mutant *KRAS*, or *ALK*-translocated versus mutation-free. Further, a prognostic classifier was developed based on Let-7d-5p and miR-769-5p expression levels that can predict overall survival [21].

## 4. Effect of miRNAs on the Chemosensitivity to EGFR-TKIs

EGFR is frequently activated in a wide range of solid tumors, representing an important therapeutic target [36,167]. Several EGFR-TKIs, including but not limited to erlotinib, gefitinib, afatinib, osimertinib, and icotinib showed efficient therapeutic activities in NSCLC patients harboring *EGFR* mutations [15,19,20,21,33,41,52,71,73,76,168,169,170,171,172,173]. Although primary responses to EGFR-TKIs in NSCLC patients have been shown, their effectiveness is frequently restricted by drug resistance development [10,148,150,174]. The intrinsic and acquired resistance to EGFR-TKIs might occur through numerous mechanisms, including *EGFR* T790M mutation, PI3K mutations, HER-2 amplification, MET amplification, and alteration into an SCLC phenotype resulting in the poor clinical outcomes of these agents [10,77,174]. Though other mechanisms of acquired resistance are still unclear. In recent years, a growing number of miRNAs have been associated with EGFR-TKI resistance, suggesting that miRNAs might be useful as new targets and predictive biomarkers for anti-EGFR treatment [175,176,177]. miR-133b could interact specifically with the 3’-UTR of *EGFR* mRNA as evidenced by bioinformatic analysis and luciferase reporter assay. Functionally, miR-133b transfection revealed a regulatory activity in suppressing EGFR mRNA translation. Furthermore, miR-133b transfection could modulate invasion, apoptosis and sensitivity to EGFR-TKI through the EGFR signaling pathways, specifically in EGFR-addicted NSCLC cells [178]. An in vitro study revealed that miR-1 significantly inhibited the EGFR-TKI effect, induced cytokines, including C-X-C motif chemokine ligand 10 and C-C motif chemokine ligand 5, leading to suppression of monocyte migration. These results indicate that the upregulated miR-1 might suppress the tumor immune microenvironment after developing EGFR-TKI resistance indicating miR-1 as a clinically useful marker to predict the therapeutic efficiency of immunotherapy in LADC patients with EGFR-TKI resistance [179]. MiR-200c-3p participates in EGFR-TKIs sensitivity by modifying the EMT process [74]. In A549 cells, miR-144-5p and miR-497-3p were linked with the signaling pathway of IGF-1R, where they increased the resistance to EGFR-TKIs [168]. Numerous studies emphasized the therapeutic potential of EGFR-regulated miR-21 [69,80,155]. EGFR signaling positively regulates miR-21 expression in lung carcinoma cell lines since the down-expression of miR-21 was induced by an EGFR-TKI [155]. The relationship between miR-21 expression with the acquired resistance to EGFR-TKIs was revealed in NSCLC cell lines, animal models, and advanced NSCLC patients [78,155,156,180,181]. A recent study indicated that the expression levels of miR-21 and Pten are negatively correlated in carcinoma tissues as compared to their matched normal tissues [180]. Further, a positive correlation was found between high miR-21/low PTEN expression levels and high TKI resistance with short overall survival in NSCLC patients undergoing TKI treatment. Thus, the change in miR-21-PTEN expression modulates TKI sensitivity in lung cancer cells [78,79].

The downstream signaling molecules of the EGFR pathway were investigated for their mRNA:miRNA associations, where PIK3R2 with miR-30a-5p, PLCG1 with miR-34a, JAK1 with miR-520e and miR-302b, and GRB2 with miR-27a can be considered as potential drug candidates for treating acquired drug resistance in NSCLCs [15]. Other RTK pathways are shared with the EGFR signaling pathway, including PDGFR, IGF-1R, c-MET, and Ron [15,57,58,59,60]. MiR-30a-5p directly targeting CD73 was downregulated in NSCLC, and that miR-30a-5p overexpression in NSCLC cell lines suppressed in vitro and in vivo cell proliferation, migration, invasion, and EMT phenotype via EGF signaling [70]. Thus, CD73 can affect the efficiency of EGFR-targeted therapies via miR-30a-5p in wild-type *EGFR* in NSCLC cells; yet, further studies are still needed to explore the underlying mechanisms of CD73-mediated drug resistance to improve the NSCLC treatment [70]. A negative correlation was observed between the expression levels of miR-449a and nicotinamide N-methyltransferase (NNMT), a metabolic enzyme linked to cancer, in EGFR-TKI-resistant NSCLC models. In addition, NNMT knockdown inhibited p-Akt and tumorigenesis, but re-expression of miR-449a induced PTEN and suppressed tumor growth [182]. Interestingly, the differential expression of 12 miRNAs between the EGFR-TKI-resistant and sensitive groups was identified, whose aberrant expressions are linked to lung tumorigenesis, drug resistance, and EGFR pathway regulation for patients with *EGFR* 19 deletion mutations [80]. Of these 12 miRNAs, miR-27a, miR-21, and miR-218 were confirmed to be overexpressed in the resistant group related to the sensitive group [80]. In addition, expression analysis of plasma miRNAs (76 up-regulated and 3 downregulated) was performed in NSCLC patients with del19 or L858R *EGFR* mutation [183]. Moreover, the overexpression of miR-222 and miR-155 was associated with poor prognoses, positive *EGFR* mutation, and metastasis in late-stage NSCLC, and that overexpression of miR-34 represents a biomarker for LADC cell type, multiple metastases, and LADC negative *EGFR* mutation [184]. Furthermore, miR-184 and miR-197 were up-regulated in LADC tumor tissue patients, who harbor *EGFR* mutations with brain metastasis, indicating these miRNAs as novel biomarkers differentiating the risk of brain metastasis [185]. Many studies demonstrate the role of miRNA in modulating cell sensitivity to erlotinib, gefitinib, osimertinib, afatinib, and icotinib [71,72,169,175,186,187].

### 4.1. Erlotinib

Erlotinib (Tarceva^®^) is an EGFR-TKI [188]. Although various mechanisms that drive resistance to EGFR-inhibitors have been identified, many cases have unclear mechanisms. Many research studies pointed out the implication of numerous miRNAs in modulating NSCLC sensitivity to erlotinib [20,57,58,59,60,83,84,169,186]. An exciting study by Gober et al. predicted a 13-miRNA signature for the response to the EGFR-TKI erlotinib in NSCLC cell lines and tumors and discriminated primarily from metastatic tumors [72,185,186,189]. For example, the ectopic expression of miR-200c changed sensitivity to erlotinib, EMT proteins expression, invasion, and metastasis in lung cells [186]. The EMT transcription factor, ZEB1, reveals altered expression in erlotinib-sensitive NSCLC, where many miRNA gene signatures are up-regulated. Again, the miR-145 expression significantly inhibited EGFR expression and improved the sensitivity to erlotinib. Thus, miR-145 plays a critical role in EGFR-TKIs resistance, cell proliferation, and survival [71]. Furthermore, the expression of *EGFR* mRNA was significantly reduced by miR-125a-5p (down-regulated in lung cancer) and consequently inhibited cell proliferation and efficiently triggered apoptosis [72]. Besides, the synergistic pretreatment with miR-125a-5p augmented the cytotoxic effect of erlotinib by reducing cellular proliferation, and enhanced the apoptotic effect, thus bypassing the resistance to EGFR-TKIs in lung cancer cells [72]. In KRAS- and TP53- mutant NSCLC, the treatment with miR-34a and let-7b (oncosuppressor), individually or in combination, lead to a synergistic potentiation of erlotinib’s anti-proliferative activity. These miRNAs could target oncogenic pathways beyond those inhibited by EGFR. Further, combinatorial treatment with miR-34a and let-7b caused the strongest synergy with erlotinib, suggesting that these miRNAs can efficiently target multiple cellular pathways involved in cellular proliferation and cell resistance to erlotinib [20,57,58,59,60,169]. Again, a significant synergistic interaction between miR-34a mimics and erlotinib was revealed in NSCLC cells either with primary or acquired erlotinib resistance that indicated miR-34a-centered therapy could be used to increase EGFR-TKI sensitivity [57,58,59,60,169]. Further, pretreatment with miR-145 improved lung cancer cells’ sensitivity to erlotinib synergistically. miR-145 could induce apoptosis and increase erlotinib levels in A549 NSCLC cells. The pretreatment with miR-145 reduced tumor growth by suppressing the expression of EGFR induced cell arrest of the G1/S cycle phase, and improved the sensitivity of A549, NSCLC cell line, to erlotinib [7,83,84].

Recently, the detection of miR-133b expression levels is associated with a better prognosis and identifies NSCLC patients who are expected to benefit from second and third-line therapy with erlotinib. miR-133b expression levels can be used to differentiate non-responder from responder patients to erlotinib, where the overexpression of miR-133b was associated with longer progression-free survival time of NSCLCs patients. miR-133b mimic transfection in A549 and H1299 NSCLC cell lines revealed that the decreased cell growth and altered morphology were due to higher expression of miR-133b, yet it did not affect sensitivity to erlotinib [190]. In functional experiments, miR-146a inhibited EGFR downstream signaling, repressed cell growth, promoted cellular apoptosis, and suppressed the migratory capacity in different NSCLC cell lines (H358, H1650, H1975, HCC827, and H292). Moreover, miR-146a improved the inhibition of cell proliferation upon treatment with EGFR-TKIs (erlotinib, gefitinib, and afatinib) as well as a monoclonal antibody (cetuximab). Interestingly, these effects were independent of the *EGFR* mutation status (wild type, resistance mutation, or sensitizing mutation). Hence, miR-146a is a strong prognostic biomarker and therapeutic candidate in NSCLC [85,86,87]. qRT-PCR assay unveiled higher miR-214 expression in the acquired erlotinib-resistant HCC827 (HCC827/ER) cells than in HCC827 cells and in NSCLC patients’ plasma with acquired EGFR-TKI resistance compared to those before exposure to EGFR-TKI therapy. LIM Homeobox 6 (LHX6) is a direct target gene of miR-214, and LHX6 expression was detected to be down-regulated in HCC827/ER cells. In transwell invasion assay, LHX6 overexpression reversed the increase in HCC827 cells’ invasive capacity induced by miR-214 overexpression, and the CRISPR-Cas9 system-mediated LHX6 knockdown inverse the reduced invasion of HCC827/ER cells caused by miR-214 down-regulation [173]. miR-506-3p expression was significantly reduced in erlotinib-resistant cells, in which Sonic Hedgehog (SHH), a novel target of miR-506-3p, is aberrantly activated. The ectopic miR-506-3p expression in erlotinib-resistant cells downregulated SHH signaling, inhibited vimentin expression, increased E-cadherin expression, thereby reducing EMT-mediated chemoresistance. This indicates that the miR-506/SHH axis might signify a novel therapeutic target for future *EGFR*-mutated NSCLC [191]. miR-641 overexpression was observed in human NSCLC samples and NSCLC cells with resistance to TKI than those sensitive to TKI, and this overexpression induced TKI resistance in NSCLC cells. Interestingly, miR-641 activates ERK signaling in NSCLC cells by directly targeting neurofibromatosis 1 (NF1), where NF1 overexpression or ERK silencing could block miR-641-induced NSCLC cell resistance to erlotinib treatment. In animal model experiments, the combination of miR-641 inhibition and erlotinib treatment showed significant inhibition of erlotinib-resistant-NSCLC growth, suppressed proliferation, and induced apoptosis compared to monotherapy [192,193]. This suggests that increased expression of miR-641 significantly contributes to erlotinib resistance development in NSCLC cells through activating ERK signaling by targeting NF1 and that inhibition of miR-641 may reverse the acquired resistance of NSCLC cells to erlotinib treatment [193].

Both primary (EGFR wild-type) and acquired resistance to EGFR-TKIs in NSCLC cells can be overcome by combined treatment with miR-34a [58,60,62,63,194]. For T790M mutants, the synergy effects may exert through miR-34a-dependent repression of AXL or MET, major mediators of erlotinib resistance, or their respective ligands, that contribute to delay resistance in *EGFR*-mutated HCC827-RC2 cells, which lack the secondary T790M mutation [195,196,197,198].

### 4.2. Gefitinib

Gefitinib (Iressa) is an EGFR-TKI that inhibits the TK activity by competitively blocking the ATP binding site. In preclinical studies, gefitinib has shown a potent activity in many tumor models, including several lung cancer cell lines and xenografts [199,200,201,202,203]. Emerging evidence indicates that various miRNAs could modulate tumor progression in response to gefitinib therapy [78,175,204,205,206,207,208,209,210]. The EGFR-independent activity of the PI3Ks/Akt or Ras/ERK pathway contributes to gefitinib-resistance in NSCLC cell lines. Besides, the anti-EGFR treatment in combination with particular inhibitors of these pathways may result in further cytotoxic effects in NSCLC cell lines [204]. The expression levels of miR-155 and miR-200c were reduced in HCC827 cells treated with gefitinib treatment. The decrease of miR-155 and miR-200c levels might be linked with either histone modifications and the EMT or the gefitinib sensitivity reduction independent of *EGFR* mutation [175,205,206,207]. Notably, miR-200a is downregulated and directly targets *EGFR* and *c-Met* mRNAs in NSCLC cells. Interestingly, miR-200a overexpression downregulates *EGFR* and *c-Met* levels and effectively inhibits invasion, migration, and gefitinib resistance in NSCLC cells [1,208,209,210,211]. MiR-30a-5p can target *EGFR* and *IGF-1R* signaling pathways, and its overexpression could regulate PI3K/AKT signaling pathway to promote cell apoptosis, impede cell migration and invasion properties, and attenuate the EGFR-inhibitor gefitinib resistance in NSCLC cell lines [15,73]. Additionally, the upregulation of miR-762 in NSCLC tissues and cells showed gefitinib resistance, indicating post chemotherapy’s poor prognosis. Notably, IL-6 signaling-induced miR-762 upregulation improved cell survival and retained NSCLC cell resistance to gefitinib [75]. Besides, the oncogenic effect of miR-762 was mediated mostly via post-transcriptional repression of ABR in gefitinib-resistant NSCLC cells [75]. In H460 and A549 cells, miR-126 restoration significantly suppressed cell growth in both cells, where the inhibition of Akt and ERK activation inhibited cell proliferation [19]. Furthermore, the overexpression of miR-126 enhanced gefitinib-induced cytotoxicity in lung cancer cells [19,212,213,214].

MiRNA microarray screening showed the significant downregulation of miR-138-5p in PC9GR cells, yet its re-expression increased the sensitivity to gefitinib in gefitinib-resistant NSCLC cell lines (H1975 and PC9GR) [76]. Further, miR-138-5p expression was reduced in gefitinib-resistant cells relative to sensitive cells. Additionally, a luciferase reporter assay and bioinformatics analysis revealed a direct target of miR-138-5p, namely GPR124, whose expression was repressed on mRNA and protein levels in NSCLC cells and LADC specimens, increasing their gefitinib-associated sensitivity [76,215,216]. Again, miR-134 and miR-487b overexpression altered the resistance to gefitinib and induced the EMT phenomenon, yet these miRNAs knockdown increased sensitivity to gefitinib via TGFβ1 and suppressed the EMT process [124]. The cluster of miR-134/miR-487b/miR-655 showed the aforementioned effects through direct targeting of MAGI2, leading to a loss of PTEN stability in NSCLC resistant to EGFR-TKI [9,78,124,217,218]. Overall, these data show that miRNAs can modulate the EGFR-TKI effect by increasing their sensitivity or resistance depending on EGFR and EGFR-activated downstream signaling molecules (Figure 2).

miR-17 family expression was upregulated, while let-7 family expression was downregulated in PC9/GR cells relative to gefitinib-sensitive PC9 cells that promoted gefitinib resistance via regulating the self-renewal ability of NSCLC cells. Importantly, miR-17 participated in cell cycle regulation by controlling its target gene cyclin-dependent kinase inhibitor 1A (CDKN1A), to sustain the proliferative potential. However, let-7 maintained stem cell characteristics by regulating the target gene MYC to retain the undifferentiated status [219]. Of note, dual inhibitors of IGF1R and EGFR markedly decreased p-AKT and p-ERK expression levels compared with the control group in H1650GR (H1650-acquired gefitinib-resistance), H460, and H1975 cell lines. miR-30a-5p mimics, along with repressing PI3K expression, can induce cell apoptosis, suppress cell invasion and migration in the treated H1650GR cell line [220,221,222,223,224]. Compared to gefitinib-sensitive cell line PC9, miR-133a-3p was significantly downregulated in PC9/GR cell line, and miR-133a-3p overexpression increased the NSCLC cells’ sensitivity to gefitinib and vice versa. Moreover, sperm-associated antigen 5 (SPAG5), an important target gene of miR-133a-3p, reversed the miR-133a-3p-mediated sensitivity of NSCLC cells to gefitinib, implying that the miR-133a-3p/SPAG5 axis played a key role in acquired resistance to gefitinib in NSCLC cells [225]. In *EGFR*-mutant NSCLC models with acquired gefitinib resistance, miR-483-3p mimics efficiently improved sensitivity of gefitinib-resistant NSCLC cells to gefitinib by suppressing proliferation and promoting apoptosis. Also, miR-483-3p reversed EMT and repressed migration, invasion, and metastasis of GR NSCLC cells. Mechanistically, miR-483-3p directly targeted integrin β3, consequently inhibiting the downstream FAK/Erk signaling pathway, highlighting miR-483-3p is a prospective target for combination treatment to overcome acquired EGFR TKI resistance in EGFR-mutant NSCLC [226,227]. miR-625-3p can potentially target AXL receptor tyrosine kinase, and this miRNA expression is markedly decreased in the HCC827GR cell line, while its overexpression partly reversed gefitinib resistance. Mechanistic analysis indicated that TGF-β1-induced EMT might contribute to the miR-625-3p/AXL signaling that mediated gefitinib resistance [228].

### 4.3. Other EGFR-TKI

Clinical analysis revealed a significantly higher miR-146b-5p expression in pleural effusions-isolated lung cancer cells from treatment-naive patients than acquiring resistance patients to EGFR-TKI treatment [172,229,230,231,232]. Further, ectopic expression of miR-146b-5p improved EGFR-TKI-induced apoptosis in EGFR-TKI-resistant cells, EGFR-independent and -dependent osimertinib-resistant primary cancer cells (PE2988 and PE3479). In rescue experiments, miR-146b-5p target IRAK1 and in turn, repressed NF-κB activity and NF-κB-related IL-6 and IL-8 production. Thus, miR-146b-5p/IRAK1/NF-κB signaling promotes EGFR-TKI resistance [172]. EMT phenomenon was indicated in HCC827-OR and PC-9-OR cells. Microarray and qRT-PCR analyses unveiled the co-upregulated miR-210-3p in exosomes isolated from HCC827-OR and PC-9-OR cells relative to those isolated from parental HCC827 and PC-9 cells. HCC827-OR cell-derived exosomes provoked EMT changes and osimertinib resistance in HCC827 cells. As a result, miR-210-3p induction directly mediated the EMT phenomenon and resistance to osimertinib in HCC827 cells [170,233,234,235,236,237]. Using the GEO database to analyze miRNA array data and the interaction networks, miR-184, miR-30d-3p, miR-542-3p, and miR-500a were shown to suppress EGFR-TKI, confirming their chemo-resistant mechanism [122]. A reversible tolerant state to osimertinib, an EGFR inhibitor, was driven by miR-147b, the foremost overexpressed miRNA in both *EGFR*-mutated and osimertinib-tolerant lung cancer cells, by suppressing succinate dehydrogenase and VHL that linked to the pseudohypoxia and tricarboxylic acid cycle (TCA) pathways [77]. Using the differential lncRNA expression and transcriptome sequencing analyses, APCDD1L-AS1, a novel lncRNA, was the most upregulated lncRNA in icotinib-resistant LAD cells promoted icotinib resistance and upregulated the EGFR protein expression level by sponging with miR-1322/miR-1972/miR-324-3p to remove the transcription inhibition of SIRT5 [238,239]. Moreover, SIRT5 increased EGFR expression and activation by suppressing EGFR autophagic degradation, thus promoting icotinib resistance. Consistently, the autophagy initiator rapamycin could decrease EGFR levels and enhance the sensitivity of icotinib-resistant LAD cells to icotinib [171].

## 5. Conclusions

Collectively, the EGFR signaling pathway and dysregulated miRNA expression pattern are key players in NSCLC development and treatment response. MiRNA–EGFR crosstalk can induce or suppress tumor growth based on the expressed miRNAs’ oncogenic or tumor-suppressive functions. The signature of miRNA/EGFR expression or miRNA–EGFR interactions can be utilized as diagnostic, prognostic, and therapeutic biomarkers in NSCLCs. Therefore, the miRNA–EGFR regulatory network can control many cellular processes. miRNA-dependent EGFR expression and/or hyperactivation have been associated with lung cancer cell behavior, including cell survival, resisting cell apoptosis, cell migration and invasion, proliferative activity, and modulating cell sensitivity to TKIs. However, these cellular processes are cell-context-dependent, e.g., NSCLC subtypes (LADC, LSCC, or LLCC). Interestingly, the use of EGFR-TKIs has considerably improved the prognosis of NSCLC patients harboring *EGFR* mutations. However, drug resistance development, either de novo or acquired, restrains the EGFR-TKI efficacy for prolonged usage (no longer than one year). Thus, these patients show enhanced disease progression and shorter survival. Furthermore, in a few *EGFR*-mutated NSCLC cases, the intrinsic mechanisms of drug resistance that exist before TKI drug treatment preclude its clinical benefit. The exploration of miRNA-dependent EGFR signaling that affects lung cancer cell behavior will essentially improve the design of a therapeutic modality for NSCLC. The integration of lung cancer screening algorithms into miRNA-dependent EGFR expression may be a useful tool for improving the screening specificity and lowering the mortality rate associated with lung cancer. Moreover, identifying potential predictors for the response to EGFR-TKIs therapies will contribute to the selection of patients with NSCLC who benefit from treatment and minimize the side effects of ineffective therapy exposure.

## Figures and Tables

**Table 1:** Role of miRNAs via targeting EGFR or its downstream signaling in modulating lung cancer cell behavior in preclinical models and clinical specimens.

Type of miRNA	Proposed Mechanism of Action in Lung Cancer	Preclinical and Clinical Studies	Methodology	Reference
Oncosuppressor miRNAs				
miR-27a	- Regulation of MET and EGFR axis	A549, H1299, and CALU-1 NSCLC cell lines	qRT-PCR Western blot Luciferase reporter assay	[52]
miR-133a	- Suppressing EGFR, p-ERK, and p-AKT - Augmenting caspase-3 protein expression - Inducing apoptosis; repressing cell growth	Human NSCLC tissues and adjacent normal lung tissue H358 human NSCLC cell line transfected with miR-133a mimics	qRT-PCR Immunohistochemistry (IHC)	[64,65,66]
miR-25-3p	- Prognostic biomarker in NSCLC by regulating TGFβ and EGFR signaling	miRNA regression model supported by target prediction databases	Multiple linear regression based on expression levels	[8]
miR-128b	- Regulating the expression of EGFR	Human NSCLC and adjacent normal lung tissue samples	qRT-PCR Semi-quantitative RT-PCR IHC analysis	[49]
miR-134	- Repressing EGFR-related signaling pathways - Inhibiting NSCLC cell proliferation by promoting apoptosis and/or cell cycle arrest	NSCLC cell lines (A549, H1299, H520, and H1975)	qRT-PCR MTT assay Flow cytometry Luciferase reporter assay RNAi and rescue experiments A549 xenograft in nude mice	[36]
miR-34a	- Suppressing tumor growth and metastasis and promoting cell apoptosis - Targeting many cellular pathways, including cell proliferation, erlotinib resistance, and EGFR-inhibited pathways	Human NSCLC and adjacent normal lung tissue samples A549 (EGFR-wild type), SPC-A1 and HCC827 (EGFR-mutated) cell lines lung carcinoma xenograft mouse model	qRT-PCR Cell proliferation assay Cell transwell assay Luciferase reporter assay Western blot analysis xenograft assay IHC analysis	[20,57]
miR-542-5p	- Associated with EGFR, vascular invasion, advanced TNM stage, lymphatic metastasis, and patients’ poor prognosis	Human NSCLC and adjacent normal lung tissue samples	qRT-PCR	[67]
miR-146a-5p	- Targeted EGFR and NF-κB signaling - Decreasing cellular expression and release of CCL2, a chemokine	A549 cells	RNA-Seq Gene ontology analysis qRT-PCR	[68]
miR-183 miR-210 miR-34c	- Correlated with lymphovascular invasion - Independently associated with T stage - High expression exhibited poor overall survival in the exon 19 mutated EGFR group - All 3 miRNAs were related to poor tumor differentiation	Human mutated LADC and adjacent normal lung tissue samples	Microarray analysis qRT-PCR	[25]
miR-125b	- Predicting EGFR mutational status and gefitinib-sensitivity - Associated with disease-free survival and overall survival	Human NSCLC and adjacent normal lung tissue samples and plasma gefitinib-sensitive PC9, and gefitinib-resistant A549 and H1299 human lung ADC cells	MicroRNA array Genotyping of EGFR mutational status	[69]
miR-30a-5p	- Suppressing cell proliferation, migration, invasion, and EMT	Human NSCLC and adjacent normal lung tissue samples lung carcinoma xenograft mouse model	CCK-8 and clonogenic assays Wound healing, migration and invasion assays	[70]
miR-145	- Inhibiting EGFR expression - Improving the sensitivity to erlotinib - Cell proliferation and survival - Inhibition of cell growth in the EGFR mutant lung adenocarcinoma - Induced cell arrest of G1/S cycle phase	NSCLC cell line A549	qRT-PCR Trypan blue and MTT assays ELISA cell death assay Combination effect analysis	[71]
miR-200a	- Downregulating EGFR and c-Met levels and inhibited invasion, migration, and gefitinib resistance	lung cancer cell lines H3255 (L858R EGFR allele), H1975 (L858R/T790M mutations in EGFR), and HCC827	qRT-PCR Luciferase assays Western Blot Wound-Healing Assay Cell Invasion Assay MTS assay and BrdU incorporation assay	[1]
miR-125a-5p	- Downregulating EGFR mRNA expression - Inhibiting cell proliferation and triggered apoptosis - Augmenting the erlotinib’s cytotoxic effect reduced cellular proliferation and enhanced its apoptotic effect	A549 lung cancer cells	qRT-PCR Trypan blue assays MTT assay Combination index (Chou-Talalay) ELISA cell death assay kit	[72]
miR-30a-5p	- Targeting EGFR and IGF-1R signaling pathways - Regulating PI3K/AKT signaling pathway - Promoting cell apoptosis, impeding cell migration and invasion properties, and reducing gefitinib resistance	Gefitinib-resistant NSCLC cell lines, H460 and H1975	Western Blot Annexin V-FITC Apoptosis Detection Kit CytoSelect™ Cell Invasion Assay Kit Wound healing assay	[15,73]
let-7b	- Targeting many cellular pathways, including cell proliferation, and EGFR-inhibited pathways - Potentiation of erlotinib anti-proliferative activity	NSCLC cells bearing clinically relevant mutations in KRAS and TP53 (H358, H23, H441, Calu-6) or NRAS and TP53 (H1299)	qRT-PCR Sulforhodamine B (SRB) assays	[20,49]
miR-200c-3p	- Increasing sensitivity to EGFR-TKIs in EGFR mutant NSCLC by modifying the EMT process	EGFR-mutant NSCLC and adjacent normal lung tissue EGFR TKI-sensitive cell lines- PC9 and HCC287, and gefitinib-resistant PC9/gef cells that harbor a deletion in exon 19 of EGFR	MicroRNA array qRT-PCR Western Blot Migration assay Cytotoxicity and apoptosis assays	[74]
miR-762	- Gefitinib resistance and poor prognosis of post-chemotherapy - IL-6 signaling-induced miR-762 upregulation improved cell survival and retained NSCLC cells’ resistance to gefitinib	NSCLC Cell lines with EGFR mutations PC-9 (E746-A750 del) NCI-H820 (E746-E749 del) NCI-H1650 (E746-A750 del) NCI-H1975 (L858R) A549, NCI-H2170, NCI-H1993, NCI-H2126, NCI-H1299, NCI-H1648, NCI-H1703 and NCI-H2347 (WT) lung carcinoma xenograft mouse model	qRT-PCR Cytotoxicity *In vivo* chemosensitivity Luciferase reporter assay	[75]
miR-126	- Suppression of Akt and ERK activation - Suppressing cell growth; inhibited cell proliferation - Enhanced gefitinib-induced cytotoxicity	NCI-H460 (H460) and A549 cells lung carcinoma xenograft mouse model	qRT-PCR Growth inhibition assay Western Blot	[19]
miR-138-5p	- Increasing the sensitivity to gefitinib	Human NSCLC and adjacent normal lung tissue samples NSCLC cell lines PC9 and H1975	qRT-PCR Luciferase assays Western Blot IHC	[76]
Oncogenic miRNAs				
miR-147b	- Inducing tolerant-state to osimertinib in both EGFR-mutated and osimertinib-tolerant lung cancer cells - Suppressing succinate dehydrogenase and VHL that linked to the pseudohypoxia and TCA pathways	EGFR-wild type cell lines H358, H460, A549, H1299, and H69 (ATCC) EGFR-mutant cell lines H1650, H1975, HCC827, HCC827GR, PC9, PC9ER, and H3255 Patient-derived Xenograft Tumor Specimens	MicroRNA array Colony Formation Assay High-Throughput Sequencing Western Blot H&E Staining and Immunofluorescence. Targeted Mass Spectrometry.	[77]
miR-21	- Reinforcing the aberrant regulation linked to lung carcinogenesis in never-smokers - More dramatic expression changes in EGFR-mutated patient mutations as opposed to EGFR wild-type cases - A strong association between p-EGFR and miR-21 levels and the suppression of miR-21 by the EGFR-TKI AG1478	- Data set - NSCLC patients with EGFR 19 deletion - Another cohort with EGFR 19 deletion mutations, who had dramatically different responses to EGFR-TKI (for miRNA expression validation)	Gene expression data (target scan database) qRT-PCR	[78,79,80]
Oncogenic/Oncosuppressor miRNAs				
miR-29b	- Targeting *TNFAIP3/A20* (NF-κB negative regulator) - miR-29b–refractory isoform of TNFAIP3 restored NF-κB and extrinsic apoptosis - confers sensitivity to intrinsic apoptosis induced by cisplatin exposure.	mutated *KRAS*^G12V^ Cells	Microarray analysis Mechanistic investigations	[41]
miR-7	- Enhanced phosphorylation of c-Myc and EGFR in EGFR mutant (L858R), (CL1-5 cells), promoting miR-7 expression - EGFR prompts miR-7 expression via Ras/ERK/Myc pathway - Reducing the ERF level; inducing cell growth and tumor formation; raising the mortality rate - Repressing cell proliferation, tumorigenicity; tempting cell apoptosis - Downregulating EGFR and RAF-1 expression	*EGFR*-silenced cells *EGFR* mutant (L858R) CL1-5 cells.	MiRNA microarray analysis qRT-PCR	[81,82]

## Data Availability

Not applicable.

## Abbreviation

ABR	ABR Activator of Rho GEF and GTPase
ADCY1	Adenylate Cyclase 1
ADCY6	Adenylate Cyclase 6
AKT	Protein kinase B
ALK	Anaplastic Lymphoma Kinase
AT1	Angiotensin II type 1
cAMP	cyclic adenosine -3’,5’-monophosphate
CCL2	C-C Motif Chemokine Ligand 2
CCNE1	cyclin E
CDK4	Cyclin-Dependent Kinase 4
CDKN1A	Cyclin-Dependent Kinase Inhibitor 1A
CTNNB1	catenin beta 1
CDR1as	Circular RNA CDR1as
c-MET	hepatocyte growth factor receptor
DUSP4	Dual Specificity Phosphatase 4
EGF	Epidermal Growth Factor
EGFR	Epidermal Growth Factor Receptor
eIF4e	Eukaryotic translation initiation factor 4E
EMT	Epithelial-Mesenchymal Transition
ERF	Ets2 transcriptional repression factor
ERK1/2	Extracellular Signal-Regulated Kinase 1/2
GABBR1	Gamma-Aminobutyric Acid Type B Receptor Subunit 1
GIPR	Gastric Inhibitory Polypeptide Receptor
GPR124	G protein-coupled receptor124
GRB2	growth factor receptor-bound protein 2
HIF-1α	Hypoxia Inducible Factor 1 Subunit Alpha
HuR	Human Antigen R
IC50	Inhibit Cellular Proliferation by 50%
IGF-1R	Insulin-like Growth Factor Receptor-1
IL-6	Interleukin-6
JAK1	Janus kinase 1
KRAS	Kirsten Rat Sarcoma Viral Oncogene Homolog
LADC	Lung Adenocarcinoma
3LL	Lewis lung cancer
LSCC	Lung Squamous Carcinoma
MAGI2	Membrane Associated Guanylate Kinase, WW And PDZ Domain Containing 2
MAPK (MEK)	Mitogen-Activated Protein Kinase
mTOR	mammalian/mechanistic target of rapamycin.
MUC4	Mucin 4
NF-kB	Nuclear Factor Kappa-Light-Chain-Enhancer of Activated B Cells
NSCLC	Non-Small Cell Lung Cancer
NRSF	Neuron-Restricted Silencing Factor
NUDT-1	Nucleoside X-type Motif-1
PDE4B	Phosphodiesterase 4b
PDE4C	Phosphodiesterase 4c
PDGFRa	platelet-derived growth factor receptor Alpha
PI3K	Phosphatidylinositol 3-kinase
PIK3CD	Phosphatidylinositol-4,5-Bisphosphate 3-Kinase Catalytic Subunit Delta
PIK3R2	phosphoinositide-3-kinase regulatory subunit 2
PLCG1	phospholipase C gamma 1
PTEN	phosphatase tensin homologue
PTK2	protein tyrosine kinase 2
SCLC	Small Cell Lung Cancer
STAT	Signal Transducer and Activator of Transcription
RAAS	Renin-Angiotensin-Aldosterone System
Ras	Rat Sarcoma
Raf	Rapidly Accelerated Fibrosarcoma
RAF-1	Rapidly Accelerated Fibrosarcoma homolog-1
RHOA	Transforming protein RhoA
Ron	a protein tyrosine kinase related to c-MET
RTK	Receptor Tyrosine Kinase
TCA	Tricarboxylic Acid Cycle
TGFβ	Transforming Growth Factor Beta
TKIs	Tyrosine Kinase Inhibitors
TNFAIP3	Tumor necrosis factor, alpha-induced protein 3 or A20
TNM	Tumor, nodes, and metastasis
TRPM2	Transient receptor potential cation channel, subfamily M, member 2
VEGFA	Vascular endothelial growth factor-A
VHL	Von Hippel–Lindau disease
ZEB1	zinc finger E-box binding homeobox 1
